# Negativity of the Casimir Self-Entropy in Spherical Geometries

**DOI:** 10.3390/e23020214

**Published:** 2021-02-10

**Authors:** Yang Li, Kimball A. Milton, Prachi Parashar, Lujun Hong

**Affiliations:** 1 Department of Physics, Nanchang University, Nanchang 330031, China; leon@ncu.edu.cn; 2 Homer L. Dodge Department of Physics and Astronomy, University of Oklahoma, Norman, OK 73019, USA; 3 John A. Logan College, Carterville, IL 62918, USA; Prachi.Parashar@jalc.edu; 4 Institute of Space Science and Technology, Nanchang University, Nanchang 330031, China; ljhong@ncu.edu.cn

**Keywords:** Casimir free energy, entropy, Abel–Plana formula

## Abstract

It has been recognized for some time that, even for perfect conductors, the interaction Casimir entropy, due to quantum/thermal fluctuations, can be negative. This result was not considered problematic because it was thought that the self-entropies of the bodies would cancel this negative interaction entropy, yielding a total entropy that was positive. In fact, this cancellation seems not to occur. The positive self-entropy of a perfectly conducting sphere does indeed just cancel the negative interaction entropy of a system consisting of a perfectly conducting sphere and plate, but a model with weaker coupling in general possesses a regime where negative self-entropy appears. The physical meaning of this surprising result remains obscure. In this paper, we re-examine these issues, using improved physical and mathematical techniques, partly based on the Abel–Plana formula, and present numerical results for arbitrary temperatures and couplings, which exhibit the same remarkable features.

## 1. Introduction

It is ordinarily expected that entropies of closed systems should be positive. This follows from the Boltzmann definition in terms of the number of microstates Ω, so the entropy is given as $S=k_{B}\ln\Omega$ ($k_{B}$ is the Boltzmann constant). Quantum-mechanically, in terms of the density operator ρ, the entropy is $S=-k_{B}\mathrm{Tr}\rho \ln\rho$. However, there are intriguing possibilities of negative entropy [1,2,3,4,5].

Here we are considering quantum-fluctuational or Casimir free energies and entropies. For two parallel conducting plates possessing nonzero resistivity, the entropy corresponding to the interaction free energy vanishes at zero temperature, as required by the Nernst heat theorem (third law of thermodynamics). However, for sufficiently low temperatures, compared to the inverse of the plate separation, a region of negative interaction entropy emerges [6]. However, the expectation at that time was that the total entropy must be positive. Negative Casimir interaction entropies also occurred without dissipation between a sphere and a plane [7,8,9,10], both perfectly conducting, between two perfectly conducting spheres [11,12], or between an atom and a “plasma-sphere” (see below) [13]. This was systematically explored in the dipole regime [14,15].

However, indeed, it turned out that the sphere-plane problem was resolved by considering the self-entropy of the plate and the sphere separately. The former vanishes in the perfectly conducting limit, but the latter is just such as to cancel the most negative contribution of the interaction entropy [16,17]. The sphere–sphere entropy is then seen to be clearly positive as well.

Going beyond the case of a perfectly conducting sphere has proved to be more subtle. We carried out a systematic treatment for an imperfectly conducting sphere, modeled by a δ-function sphere, or a “plasma-sphere,” described by the potential $V=\varepsilon -1=\lambda (1-\hat{r}\hat{r})$ (in terms of polar coordinates based on the center of the sphere), where the transversality condition is required by Maxwell’s equations. We take the coupling λ to be frequency-dependent, according to the plasma model, $\lambda =\lambda_{0}/\left(\zeta^{2}a\right)$, where ζ=−iω is the Euclidean frequency, and *a* is the radius of the sphere. The dimensionless coupling constant $\lambda_{0}$ is necessarily positive. In the limit of λ→∞, we recover the entropy for a perfectly conducting sphere first obtained by Balian and Duplantier [18]. However, for a sufficiently weak coupling, even at high temperatures, we found that the entropy could turn negative [19,20]. (The results found there largely agreed with those found subsequently by Bordag and Kirsten [21,22].)

Since the transverse electric contribution to the entropy is always negative and presents no difficulties in its evaluation, in this paper we concentrate on the transverse magnetic free energy, $F_{H}$. One feature of the analysis here is that we always subtract an infrared-sensitive, but unphysical term, which we only subtracted in an *ad hoc* manner in Ref. [19]. The most salient element of our new treatment, however, is the emphasis on the Abel–Plana formula and the numerical computations based upon that formulation. In the next section, we give the general formulas for this model and recast the result in Abel–Plana form, which expresses the finite temperature-dependent part of the free energy in terms of a mode sum over the phase of a quantity involving spherical Bessel functions. Afterwards, in Section 3, we specialize to weak coupling, where the mode sum can be carried out explicitly for the lowest-order term. The result agrees with that found in Ref. [19]. The low-temperature limit is considered in Section 4; we extract coincident free energies using both the Euclidean and the (real-frequency) Abel–Plana formulations. We briefly review the previous result for high temperatures in Section 5. Finally, we present general numerical results in Section 6, which, for coupling and temperature of order unity (in units of 1/a) turn out to be remarkably similar to those found for low temperature. Further numerical explorations have shown how the analytic asymptotic behaviors are realized. Concluding remarks round out the paper.

In this paper, we adopt natural units, with $\hbar =c=k_{B}=1$.

## 2. Transverse Magnetic Free Energy of Plasma-Shell Sphere

We concentrate on the transverse-magnetic (TM) contribution to the free energy of a δ-sphere, since the transverse electric (TE) part seems unambiguous and always yields a negative contribution to the entropy. As derived in Ref. [19], the TM free energy is given by

(1) $$F_{H}=\frac{T}{2}\sum_{n=-\infty }^{\infty }e^{in\alpha \tilde{\tau }}\sum_{l=1}^{\infty }\left(2l+1\right)P_{l}\left(\cos\delta \right)\ln\left[1-\lambda_{0}\frac{\alpha |n|e_{l}^{\prime }\left(\alpha |n|\right)s_{l}^{\prime }\left(\alpha |n|\right)}{\alpha^{2}n^{2}+{\tilde{\mu }}^{2}}\right],$$

where $\tilde{\tau }=\tau /a\to 0$ is the dimensionless time-splitting regulator, δ→0 is the angular point-splitting regulator, and α=2πaT, so that $n\alpha =a\zeta_{n}$, where $\zeta_{n}$ is the Matsubara frequency. Further, we have inserted an infrared regulator $\tilde{\mu }=\mu a$, modeled as a photon mass. Here the modified Riccati–Bessel functions are

(2) $$s_{l}\left(x\right)=\sqrt{\frac{\pi x}{2}}I_{l+1/2}\left(x\right),\;e_{l}\left(x\right)=\sqrt{\frac{2x}{\pi }}K_{l+1/2}\left(x\right).$$

We might hope to eliminate the $\tilde{\mu }$ regulator dependence, formally, by subtraction of an unphysical coupling-independent term:

(3) $$\begin{array}{ccc} F_{H} & = & \frac{\alpha }{2\pi a}\sum_{n=0}^{\infty }{}^{\prime }\cos\left(n\alpha \tilde{\tau }\right)\sum_{l=1}^{\infty }\left(2l+1\right)P_{l}\left(\cos\delta \right)\left(\ln\left[{\left(n\alpha \right)}^{2}+{\tilde{\mu }}^{2}-\lambda_{0}f_{H}\left(l,n\alpha \right)\right]-\ln\left[{\left(n\alpha \right)}^{2}+{\tilde{\mu }}^{2}\right]\right), \end{array}$$

where the prime on the summation sign means that the n=0 term is to be counted with half weight, and we have abbreviated $f_{H}\left(l,x\right)=xe_{l}^{\prime }\left(x\right)s_{l}^{\prime }\left(x\right)$. The subtracted term was evaluated in Ref. [19], because $\sum_{l=1}^{\infty }\left(2l+1\right)P_{l}\left(\cos\delta \right)=-1$:

(4) $$F_{H}^{\mathrm{sub}}=\frac{T}{2}\sum_{n=-\infty }^{\infty }e^{in\alpha \tilde{\tau }}\ln\left[n^{2}\alpha^{2}+{\tilde{\mu }}^{2}\right]=-\frac{1}{2\tau }+T\ln\frac{\mu }{T}.$$

We discarded this term as unphysical (it makes no reference to the properties of the sphere) frequently throughout ref. [19], although it was not done systematically. Now we propose doing so. We can then recast the remainder of $F_{H}$ using the Abel–Plana formula, which reads

(5) $$\sum_{n=0}^{\infty }{}^{\prime }g\left(n\right)=\int_{0}^{\infty }dt\;g\left(t\right)+i\int_{0}^{\infty }dt\frac{g(it)-g(-it)}{e^{2\pi t}-1}.$$

Applied to Equation (3) after the omission of the subtracted term (4), we see that the first integral gives a contribution independent of *T*, which is the (divergent) zero-temperature TM energy of the sphere [23]. We are here only concerned with the temperature-dependent part, which we can rewrite as

(6) $$\Delta F_{H}=-\frac{1}{\pi a}\int_{0}^{\infty }\frac{dx}{e^{2\pi x/\alpha }-1}\sum_{l=1}^{\infty }\left(2l+1\right)\arg\left[-x^{2}-\lambda_{0}f_{H}\left(l,ix\right)\right].$$

Here, we have dropped the regulators because this expression is finite.

The definition of the argument function is somewhat subtle. We choose it to be defined by the usual arctangent,

(7) $$\arg\left(z\right)=\arctan\left(\frac{ℑz}{ℜz}\right),\;-\frac{\pi }{2}<\arctan y\leq \frac{\pi }{2},$$

which is discontinuous when ℜz passes through zero. This choice is necessary in order to have a well-defined limit at zero temperature (see Section 4.2). It also guarantees that the free energy vanishes for zero coupling, which would seem to be an obvious physical requirement. Therefore, the argument appearing in Equation (6) is

(8) $$\arg\left[-x^{2}-\lambda_{0}f_{H}\left(l,ix\right)\right]=\arctan\left(\frac{\lambda_{0}\frac{\pi }{2}J_{\nu }^{2}\left(x\right)}{-x^{2}+\lambda_{0}\frac{\pi }{2}J_{\nu }\left(x\right)Y_{\nu }\left(x\right)}\right),\;\nu =l+\frac{1}{2}.$$

The functions appearing here are, in terms of ordinary Bessel functions $J_{\nu }$ and $Y_{\nu }$,

(9a) $$\begin{array}{ccc} J_{\nu }\left(x\right) & = & -\sqrt{\frac{2x}{\pi }}{\left[xj_{l}\left(x\right)\right]}^{\prime }=\left(\nu -1/2\right)J_{\nu }\left(x\right)-xJ_{\nu -1}\left(x\right), \end{array}$$

(9b) $$\begin{array}{ccc} Y_{\nu }\left(x\right) & = & -\sqrt{\frac{2x}{\pi }}{\left[xy_{l}\left(x\right)\right]}^{\prime }=\left(\nu -1/2\right)Y_{\nu }\left(x\right)-xY_{\nu -1}\left(x\right), \end{array}$$

$j_{l}$ and $y_{l}$ being the corresponding spherical Bessel functions.

The ultraviolet convergence of $\Delta F_{H}$ in Equation (6) in *x* is assured by the exponential factor, but the convergence in *l* requires further investigation. It is easily checked that

(10) $$f_{H}\left(l,ix\right)∼-\frac{\nu }{2}\;\mathrm{as}\;l\to \infty ,$$

so

(11) $$\arg[-x^{2}-\lambda_{0}f_{H}\left(l,ix\right)]\to 0,\;\mathrm{as}\;l\to \infty .$$

## 3. Weak Coupling

With the above definition of the argument function, we can readily work out the weak coupling expansion of the free energy. The leading term in $\lambda_{0}$ is obtained from the first term in the expansion of the arctangent, so

(12) $$\Delta F_{H}^{\left(1\right)}=\frac{\lambda_{0}}{\pi a}\int_{0}^{\infty }\frac{dx}{x}\frac{1}{e^{2\pi x/\alpha }-1}\sum_{l=1}^{\infty }\left(2l+1\right){\left({\left[xj_{l}\left(x\right)\right]}^{\prime }\right)}^{2}.$$

The sum on *l* can be carried out using the addition theorem for spherical Bessel functions

(13) $$\sum_{l=0}^{\infty }\left(2l+1\right)j_{l}\left(x\right)j_{l}\left(y\right)=\frac{\sin(x-y)}{x-y}.$$

Thus, the *l* sum in Equation (12) is

(14) $$\lim_{y\to x}\frac{\partial }{\partial x}\frac{\partial }{\partial y}xy\left[\frac{\sin(x-y)}{x-y}-j_{0}\left(x\right)j_{0}\left(y\right)\right]=1+\frac{x^{2}}{3}-\cos^{2}x,$$

since $j_{0}\left(x\right)=\frac{\sin x}{x}$. This yields the same result found in Ref. [19], Equation (5.34),

(15) $$\Delta F_{H}^{\left(1\right)}=\frac{\lambda_{0}}{\pi a}\int_{0}^{\infty }\frac{dx}{x}\frac{1}{e^{2\pi x/\alpha }-1}\left(\sin^{2}x+\frac{x^{2}}{3}\right)=\frac{\lambda_{0}}{4\pi a}\left[\ln\left(\frac{\sinh\alpha }{\alpha }\right)+\frac{\alpha^{2}}{18}\right],$$

found there both using the Abel–Plana (real frequency) and the Euclidean frequency formulations.

## 4. Low Temperature

### 4.1. Euclidean Frequency Argument

Let us first write the subtracted free energy in the original point-splitting form:

(16) $$\Delta F_{H}=\frac{T}{2}\sum_{n=-\infty }^{\infty }e^{ix\tilde{\tau }}\sum_{l=1}^{\infty }\left(2l+1\right)P_{l}\left(\cos\delta \right)\ln\left[x^{2}-\lambda_{0}f_{H}\left(l,x\right)\right],$$

where x=2πnaT=nα. Thus, the low temperature limit corresponds to small *x*. Using the small-argument expansion for the Bessel functions,

(17) $$\begin{array}{ccc} f_{H}\left(l,x\right) & ∼ & -\frac{l(l+1)}{2l+1}-\frac{3+2l(l+1)}{\left(4l^{2}-1\right)\left(2l+3\right)}x^{2}+O\left(x^{4}\right)-x^{2l+1}\left[{\left(-1\right)}^{l}2^{-2(l+1)}\frac{{\left(l+1\right)}^{2}\pi }{\Gamma {\left(l+3/2\right)}^{2}}+O\left(x^{2}\right)\right],\;x\ll 1, \end{array}$$

so it is seen that the leading odd term in *x* arises only from the l=1 term, where

(18) $$f_{H}\left(1,x\right)∼-\frac{2}{3}-\frac{7}{15}x^{2}+\frac{4}{9}x^{3}+O\left(x^{4}\right),\;x\ll 1.$$

Thus, the logarithm in the free energy is

(19) $$\ln\left[x^{2}-\lambda_{0}f_{H}\left(1,x\right)\right]∼\ln\frac{2\lambda_{0}}{3}+\left(\frac{3}{2\lambda_{0}}+\frac{7}{10}\right)x^{2}-\frac{2}{3}x^{3}+O\left(x^{4}\right),\;x\ll 1.$$

This is the same as Equation (6.12) of ref. [19], except that the $x^{2}$ in the logarithm there has been removed by the subtraction.

The above analysis is relevant to the low temperature behavior because that may be extracted by using the Euler–Maclaurin formula,

(20) $$F_{H}=T\sum_{n=0}^{\infty }{}^{\prime }g\left(n\right)∼T\int_{0}^{\infty }dn\;g\left(n\right)-T\sum_{k=1}^{\infty }\frac{B_{2k}}{(2k)!}g^{\left(2k-1\right)}\left(0\right).$$

Because of the subtraction, the expansion can be carried out around n=0, since the function is now analytic there. (In ref. [19] we did the expansion around n=1, and we did, in fact, remove the $F_{H}^{\mathrm{sub}}$ term, Equation (4). See Equation (6.11) there.) The integral term in Equation (20) is independent of *T*, so the leading contribution to the entropy comes from the third derivative term, allowing us to immediately obtain, as before,

(21) $$\Delta F_{H}=-\frac{2}{15}{\left(\pi a\right)}^{3}T^{4},\;aT\ll \sqrt{\lambda_{0}},1.$$

This is the well known strong-coupling low-temperature limit [18,19].

The above, of course, corresponds to a positive entropy. However, this analysis presumed that aT was the smallest scale in the problem. On the other hand, we have another parameter, $\xi =\alpha \sqrt{\frac{3}{2\lambda_{0}}}$, which could be large if $\lambda_{0}\ll \alpha^{2}$. The analysis given in Ref. [19] is unchanged and results in the formula

(22) $$\Delta F_{H}={\left(\frac{2\lambda_{0}}{3}\right)}^{2}\frac{1}{\pi a}\left[\frac{\xi^{2}}{12}-\ln\xi -ℜ\psi \left(1+\frac{i}{\xi }\right)\right],\;\alpha \ll 1,\xi ∼1.$$

**Figure 1:**
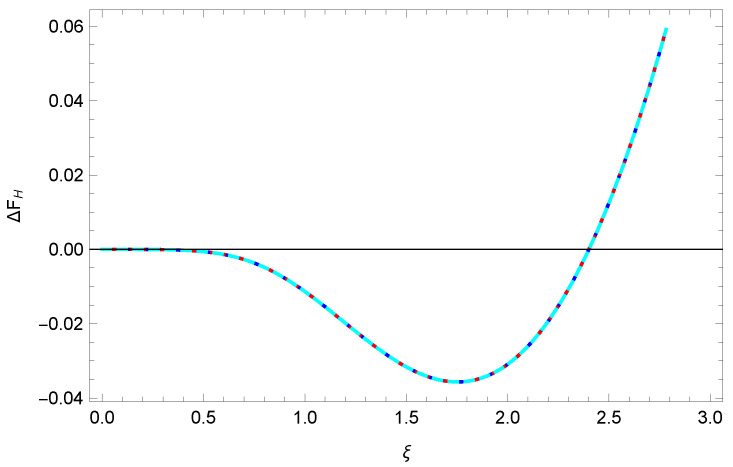
The TM free energy for low temperature in terms of $\xi =\alpha \sqrt{3/(2\lambda_{0})}$. Shown are the coincident results for the Formula (22) and for Equation (25a) with two different values of α, α=0.1 and α=0.01. Plotted is the free energy apart from a factor of ${\left(2\lambda_{0}/3\right)}^{2}/\left(\pi a\right)$. Although the slope is negative (positive entropy) for small ξ (strong coupling), it is positive (negative entropy) for large enough ξ (weak enough coupling).

Here, ψ is the digamma function. (An alternative derivation is given in Appendix A of ref. [20].) This function is plotted in Figure 3 of ref. [19] and Figure 1 of ref. [20] (see Figure 1 here). Evidently, the entropy, the negative derivative of the free energy with respect to temperature, becomes negative for sufficiently weak coupling (large ξ), as is seen from the analytic limiting behavior:

(23) $$\xi \gg 1:\;\Delta F_{H}∼\frac{2}{9}\lambda_{0}\pi aT^{2},\;\sqrt{\lambda_{0}}\ll aT\ll 1.$$

The TE contribution to the entropy is always negative, so the total entropy turns negative for a sufficiently small coupling.

### 4.2. Abel–Plana Analysis

The derivation of the same result must be achievable directly from the Abel–Plana form (6), since the Euler–Maclaurin formula is derivable from the Abel–Plana expression. It is a bit subtle, because we have to worry about the appropriate branch of the phase, but it is actually very simple.

First, we use Equation (17) with the replacement *x* by ix. (Again, the leading odd term comes from l=1.) This gives the predominant term in the phase, (x≪1, $x^{2}/\lambda_{0}∼1$)

(24) $$\arg\left[\frac{2}{3}\lambda_{0}-\left(1+\frac{7}{15}\lambda_{0}\right)x^{2}+i\frac{4}{9}\lambda_{0}x^{3}\right]=\arctan\left[\frac{\frac{2}{3}x^{3}}{1-\frac{3x^{2}}{2\lambda_{0}}}\right].$$

The TM free energy thus reads for low *T*

(25a) $$\begin{array}{ccc} \Delta F_{H} & = & -{\left(\frac{2\lambda_{0}}{3}\right)}^{2}\frac{1}{\pi a}\frac{3\xi^{3}}{\alpha^{3}}\int_{0}^{\infty }dz\frac{1}{e^{2\pi z/\xi }-1}\arctan\left[\frac{\frac{2}{3}{\left(\frac{\alpha }{\xi }\right)}^{3}z^{3}}{1-z^{2}}\right] \end{array}$$

(25b) $$\begin{array}{ccc} & \to & -{\left(\frac{2\lambda_{0}}{3}\right)}^{2}\frac{2}{\pi a}P\int_{0}^{\infty }dz\frac{1}{e^{2\pi z/\xi }-1}\frac{z^{3}}{1-z^{2}},\;\alpha \ll 1. \end{array}$$

These expressions require some explanation. For the first line, we remind the reader that, because of our choice of the branch of the arctangent to be the usual one, there is a discontinuity in the integrand at z=1, but of course this is integrable. We need, for stability, to evaluate the integral by taking a principal value there. In the second line, we replaced arctany by *y*, appropriate for small α, and the resulting singularity at z=1 is integrated by taking a principal value. Thus, numerically, both forms exactly agree with the previous Formula (22), as Figure 1 shows.

The figure shows that, for sufficiently weak coupling, the low-temperature entropy turns negative.

It is very easy (much easier than in Section 4.1) to extract the weak-coupling limit at low temperature, ξ→∞. The crucial observation is that (25b) receives contributions from only large z∼ξ when the latter is large, so the last factor in the integrand is merely −z, and the integral then gives the result (23) immediately. Note that the oddness of the arctangent around z=0 is crucial here; were there a discontinuity in the argument function at z=0, the T→0 limit would not exist.

## 5. High Temperature

We showed in Refs. [19,20] that the leading behaviors for high temperature of the TM free energy and entropy are

(26) $$F_{H}∼\frac{\lambda_{0}}{18}\pi aT^{2},\;S_{H}=-\frac{\partial }{\partial T}F_{H}∼-\frac{\lambda_{0}}{18}\alpha ,\;\alpha =2\pi aT\gg 1,\lambda_{0}.$$

Again, it is remarkable that this is first-order in the coupling. This same behavior was found in Ref. [21]. (If $\lambda_{0}\gg 2\pi aT\gg 1$, the entropy becomes positive [18].) Here, we have made the universal subtraction of the term $F_{H}^{\mathrm{sub}}$, but that should not alter the conclusion, because that contribution to the entropy is subdominant at high temperature. (Indeed, we dropped coupling-independent terms in Ref. [20].)

In ref. [20], we worked out the leading high-temperature form for the free energy starting from the Euclidean frequency expression (1) using the uniform asymptotic expansions for the Riccati–Bessel functions and the Chowla–Selberg formula. Here, it seems to be much harder to use the uniform asymptotics on the highly oscillatory real-frequency Bessel functions appearing in the Abel–Plana expressions.

## 6. Numerical Analysis

In principle, it seems that the Abel–Plana Formula (6), which is finite, should be directly evaluated to obtain the free energy for any temperature and coupling strength. (It is not possible to do so starting from the Euclidean form (16), because this still contains divergences.) The difficulty is that the phase (8) becomes an extremely oscillatory function for x>ν. Nevertheless, the sum and integral can be carried out for intermediate values of $\lambda_{0}$ and *T* with moderate computing resources.

In the numerical calculations, the behaviors of the phase in the vicinity of the singularities have to be carefully considered. When the coupling $\lambda_{0}$ is small, contributions to the free energy near these singularities are significant. Here, we have carried out the evaluations with sufficient precision to achieve reliable results, limited only by available hardware.

**Figure 2:**
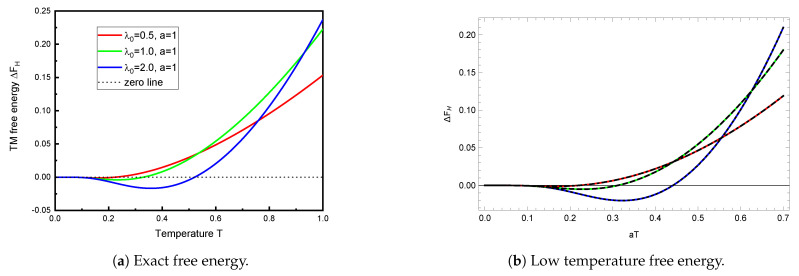
The TM free energy (in units of 1/*a*) computed from the exact Formula (6) (left panel) or the low-temperature Formula (22) or (25b) (right panel) plotted as a function of *aT* for the same intermediate values of $\lambda_{0}$, $\lambda_{0}$ = 0.5, 1, and 2, in increasing order on the right side of each figure. Although the low-temperature formula would not seem to be applicable here, since the temperature is not particularly low, it gives results that are qualitatively identical to the exact free energy seen in Figure 2a, with significant deviations apparent only at higher *T*.

Figure 2a shows the TM free energy for different moderate values of $\lambda_{0}$, as a function of temperature.

What is truly remarkable is how similar these curves are to those given by the low-temperature Formula (22), which, despite its apparent inapplicability, is shown in Figure 2b. Apparently, then, the numerical results shown in Figure 2a still largely inhabit the low-temperature regime. This is not, perhaps, so surprising, since the validity of the replacement in Equation (25b) demands aT≪1, not α≪1.

**Figure 3:**
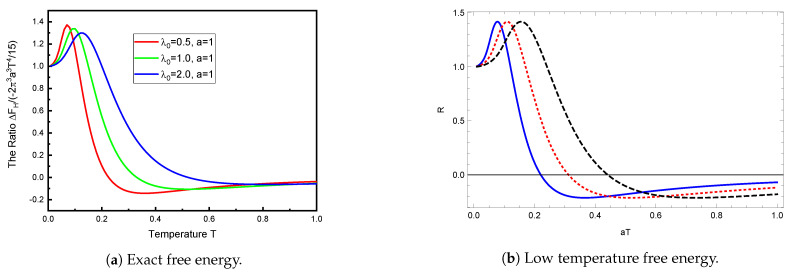
TM free energy relative to the strong-coupling low-temperature limit. The left panel shows the exact TM free energy (6) as a function of temperature *T* (in units of 1/*a*) relative to the strong-coupling low-temperature limit (21), for various values of the coupling $\lambda_{0}$. For a very low temperature, the free energy agrees with the limit (21). The nonmonotonicity is quite striking. The right panel shows the ratio *R* of Equation (22) to (21) as a function of *aT*. It is seen that the general low-temperature expression (22) captures most of the behavior shown in Figure 3a. The different curves in Figure 3b correspond to the same values of the coupling as in Figure 3a, namely, $\lambda_{0}$ = 0.5 (blue, solid), $\lambda_{0}$ = 1 (red, dotted), and $\lambda_{0}$ = 2 (black, dashed).

In Figure 3a, we compare the computed TM free energy to the strong-coupling low-temperature result (21). This is qualitatively very similar to that obtained by taking the ratio of Equations (22) and (21), as seen in Figure 3b. Again, this demonstrates that the low temperature description extends to quite large temperatures. To put this into perspective, it might help to note that aT=1 corresponds, at room temperature, to a sphere radius of a=8μm.

**Figure 4:**
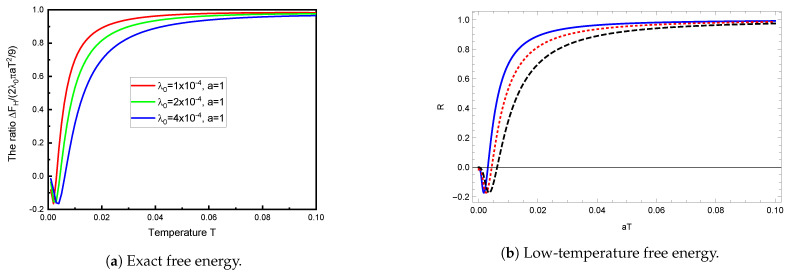
The behavior of the TM free energy for low temperatures (in units of 1/*a*), for even smaller values of the coupling, relative to the limiting value for low temperature and very small $\lambda_{0}$, Equation (23). The left panel shows the exact free energy (6), while the same ratio *R* is plotted in the right panel, except that the TM free energy is computed from the general low-temperature expression (22). The different curves are for the same values of $\lambda_{0}$ as in Figure 4a: $\lambda_{0}=10^{-4}$ (blue, solid), $\lambda_{0}=2\times 10^{-4}$ (red, dotted), and $\lambda_{0}=4\times 10^{-4}$ (black, dashed). The fact that $F_{H}$ turns negative for very small temperatures reflects the limit (21).

The weak-coupling regime for low temperature is explored in Figure 4a. The comparison here is with Equation (23). Of course, this agrees with that obtained from (22), as demonstrated in Figure 4b.

**Figure 5:**
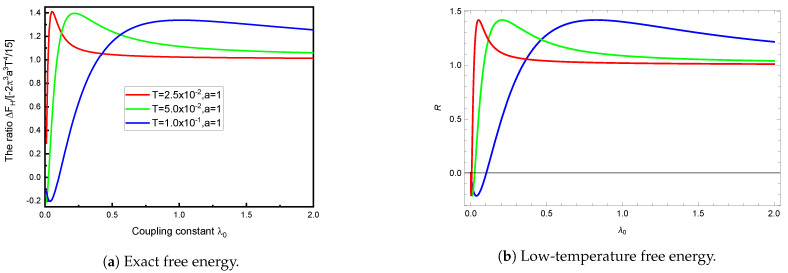
The left panel shows the ratio of the exact TM free energy (6) to the strong-coupling, low-temperature limit (21) for relatively low temperatures, as a function of $\lambda_{0}$. The reversal of sign for low $\lambda_{0}$ reflects the transition from the regime where Equation (23) applies to the strong-coupling, low-temperature limit (21). The right panel shows the same ratio, except that, instead of the exact free energy, the general low-temperature expression (22) is used for the same values of temperature. The two graphs are nearly indistinguishable. In both panels, the different curves correspond to the temperatures $a_{T}=2.5\times 10^{-2}$, $5\times 10^{-2}$, $1\times 10^{-1}$, from bottom to top on the right of each panel.

The low-temperature regime for moderate couplings is explored in Figure 5a. Again, this agrees with the low-temperature free energy (22), as shown in Figure 5b.

**Figure 6:**
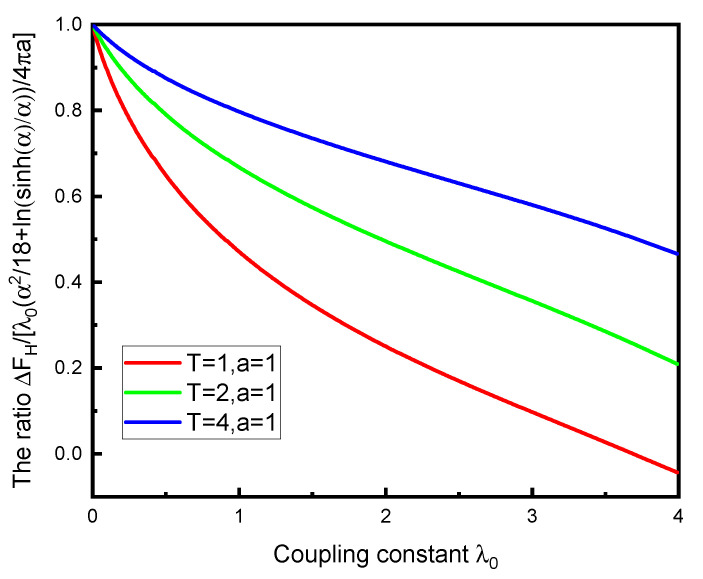
The free energy (6) compared to the $O(\lambda_{0})$ approximation (15). For small coupling, the ratio approaches unity, and the curves become flatter as temperature increases, consistent with the limiting form (26).

Finally, we compare in Figure 6 the exact free energy relative to Equation (15). We see that the weak-coupling formula is recovered as the coupling goes to zero and that the ratio tends to one as the temperature increases, consistent with Equation (26).

## 7. Conclusions

**Figure 7:**
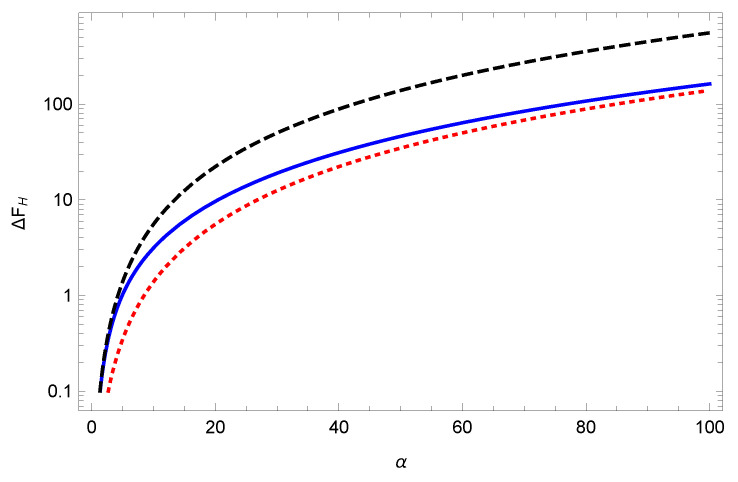
The $O(\lambda_{0})$ contribution to the free energy (blue, solid), given by Equation (15), with the prefactor $\lambda_{0}/\left(\pi a\right)$ pulled out, compared to the limiting forms (23) (low temperature, black, dashed) and (26) (high temperature, red, dotted).

In this paper, we have re-examined the question of negative entropy for a spherical plasma shell. We confirm the results first found in Ref. [19], using now a uniform subtraction of an irrelevant (infrared) divergent term, basing our re-analysis largely based on the Abel–Plana representation of the free energy. Most interesting is that the leading anomalous terms (those corresponding to negative entropy) are captured by the weak-coupling limit, which we also re-derive here. In Figure 7, we show the weak coupling TM free energy (15) compared to the low and high temperature limits, given in Equations (23) and (26), respectively. The weak-coupling contribution to the entropy is always negative.

Incidentally, it might be noted that we are not referring to the ubiquitous positive entropy of the ambient blackbody radiation. This makes no reference to the properties of the body and thus would appear to be irrelevant to our considerations.

Since the anomalous behavior seems concentrated in the $O(\lambda_{0})$ term, one might be tempted to argue that it should be subtracted from the free energy [24]. After all, at zero temperature, such terms are frequently recognized as “tadpole” terms and are often omitted as unphysical. Moreover, for a dielectric ball, at zero temperature, the “bulk subtraction” also removes automatically the linear term in (ε−1) [25]. Here, however, such a subtraction would ruin the limit to strong coupling, which has been understood for many years [18] (see, for example, Equation (21)). The analytic structure of the theory in the coupling constant is rather rigid, so *ad hoc* subtractions are not allowed. This point was made at the end of ref. [19].

In any event, the anomalous behavior is not confined to weak coupling, as the numerical analysis summarized in Section 6 shows. Therefore, the occurrence of negative entropy here is hard to deny. These remarkable findings may have profound implications for our understanding of statistical mechanics and quantum field theory.

## Data Availability

Data can be available upon reasonable request from the authors.

## Abbreviations

The following abbreviations are used in this manuscript:

TE	Transverse electric
TM	Transverse magnetic
